# Fresh Pineapple Agronomy in the Republic of Benin: Recent Trends in Calcium Carbide Use and Producer Perceptions

**DOI:** 10.1002/pei3.70026

**Published:** 2025-01-07

**Authors:** Nicodème Fassinou Hotegni, Orthia L. F. Linkpon, Charlotte A. O. Adjé, Mouizz A. B. Salaou, Enoch G. Achigan‐Dako

**Affiliations:** ^1^ Genetics, Biotechnology and Seed Science Unit (GBioS), Laboratory of Crop Production, Physiology and Plant Breeding (PAGEV), Faculty of Agricultural Sciences University of Abomey‐Calavi Cotonou Republic of Benin

**Keywords:** *Ananas comosus*, artificial flowering induction, calcium carbide (CaC_2_), cv. Sugarloaf, production system

## Abstract

Pineapple ( *Ananas comosus* (L.) Merrill) is among the main fruits produced in West Africa. This is also the case for the Republic of Benin, where pineapple fruit is regarded as an important crop for numerous producers in the Southern part of the country. However, crop production is constrained by various issues, the major one being poor yield caused by current agronomic practices. This has become a bottleneck that affects high‐quality fruit production. The objective of this research was to (i) conduct a comprehensive analysis of the current fresh pineapple production system with a focus on trends over a decade and (ii) explore the compounds used to artificially induce flowering of pineapple plants, along with the perception of fresh pineapple producers on the use of calcium carbide (CaC_2_) on their health. Semi‐structured interviews were conducted with 308 producers. Our results indicate that agronomic practices were diverse, with the involvement of more women in pineapple production over the last decade. A decrease in the involvement of young people was also observed. Cv. Sugarloaf remains the main cultivar grown, with a slight increase in cv. Smooth Cayenne. Increasing numbers of pineapple producers applied chemical fertilizers, with an increase in the use of potassium sulphate (K_2_SO_4_). Artificial flowering induction practices such as adding (i) petroleum/motor oil, (ii) cooking salt, (iii) liquid fertilizer (“Super Gro”), or (iv) urea to calcium carbide were reported, as well as health issues related to the use of calcium carbide. The research highlights the need for further studies on the use of alternatives to calcium carbide for artificial flowering induction in pineapple production in Benin.

## 1 Introduction

Pineapple ( *Ananas comosus* (L.) Merrill) is among the most consumed and traded fruits in West Africa. In that region of the continent, its production volume is estimated to be 2,881,306 tons, with a total cultivated area of 230,717 ha (FAOSTAT 2023). The main fresh pineapple‐producing countries in West Africa are Ghana, Ivory Coast, Nigeria, the Republic of Benin, and Togo (Fresh Plaza 2022). In Benin, as is the case for most countries in West Africa, pineapple is considered a crop with high export potential. It is the third most exported crop after cotton and cashew nut. Pineapple production contributes 1.2% to the country's Gross Domestic Product (GDP) and 4.3% to the agricultural GDP (Institut National de la Statistique et de l'Analyse Economique (INSAE‐Bénin) 2020). Due to increasing interest in fresh pineapple production in Benin, the land area under cultivation expanded from 116 ha in 2000 to 551 ha in 2020 (DSA 2021). Total production, which was estimated to be 51,151 tons in 2000, surged to 350,476 tons in 2020, marking an increase of > 585% over two decades (DSA 2021).

Currently, pineapple is listed among the crops to be promoted for its wide cultivation beyond the Agricultural Development Territorial Agency (ATDA) 7 zone. Despite such importance given to the production of fresh pineapple, many producers still complain about poor yield and poor access to more lucrative markets (Honfoga et al. 2020). This calls for a need to assess the fresh pineapple production system, especially given that the last assessment was conducted more than a decade ago by Fassinou Hotegni et al. (2012) and Achigan‐Dako et al. (2014).

In their study, Fassinou Hotegni et al. (2012) reported the use of calcium carbide (CaC_2_); a chemical compound primarily used in industrial and agricultural applications, known for its ability to release acetylene gas upon contact with water (Okeke et al. 2022), by all surveyed fresh pineapple producers for artificial flowering induction in pineapple plants. The product is very harmful to human health, and by extension, to the natural environment (Hussain et al. 2024; Okeke et al. 2022; Ugbeni and Alagbaoso 2023). Anani et al. (2020) reported that, in Togo, many fresh pineapple producers felt uncomfortable after using the product. Efforts have been made by the Beninese government to shift from the use of calcium carbide to a more “friendly” product named “TifBio” (activated charcoal). Despite this decision, many producers are facing challenges to produce fresh pineapple. There is therefore a need to not only reassess the current pineapple production system but also document the different flowering induction practices used. Such assessment is crucial for (i) ensuring sustainability, (ii) identifying areas for improvement, and (iii) capitalizing on emerging opportunities within this segment of the pineapple value chain.

The following questions guided this study: What has changed over the time period 2012 to 2023 in fresh pineapple production practices in the Republic of Benin? Are fresh pineapple producers aware of the risk associated with the use of calcium carbide on their health? If so, what are their perceptions of the health effects?

## 2 Materials and Methods

### 2.1 Sampling and Data Collection

In the Republic of Benin, fresh pineapple is mainly produced in the Atlantic department, where over 82% of producers are located (Kinkpe, Luckmann, and Grethe 2023; Kpenavoun Chogou et al. 2014). Within this department, fresh pineapple is mainly produced in five municipalities, namely Zè, Tori‐Bossito, Allada, Abomey‐Calavi, and Toffo. The proportions of fresh pineapple producers in these municipalities are 22.9%, 22.1%, 14.3%, 14.1%, and 7.5% in Zè, Tori‐Bossito, Allada, Abomey‐Calavi, and Toffo, respectively (Kpenavoun Chogou et al. 2014). Through analysis of statistics from the National Directorate of Agricultural Statistics in Benin, these proportions were still the same at the time we conducted this study. The five municipalities within the Atlantic department were selected for this study.

A stratified sampling method was used to select the number of fresh pineapple producers to survey in the municipalities using a two‐step approach. The number “*n*” of fresh pineapple producers to be surveyed in the whole Atlantic department was first determined, followed by the number “*n*_c_” of fresh pineapple producers to be surveyed in each municipality within the Atlantic department.

The number “*n*” of fresh pineapple producers surveyed in the Atlantic department was determined using the formula established by Dagnelie (2000):

$$n=U_{1-\alpha /2}^{2}\times \frac{P\left(1-P\right)}{d^{2}}$$

With *U*_1‐*α*/2_ = 2.452 for a threshold of *α* = 2.33%; *n* = number of pineapple producers to be sampled in the Atlantic department; *P* = percentage of fresh pineapple producers in the Atlantic department (based on data from the Directorate of Agricultural Statistics (DSA‐Benin) published in 2021, *P* was estimated to be 7.77%); *d* = the maximum admissible error fixed at 2.3%;1% ≤ *d* ≤ 15%.

The number *n* of fresh pineapple producers was calculated and estimated to be 333 for the whole Atlantic department with a 2.3% admissible error. Data from the Territorial Agency for Agricultural Development (hub 7), in charge of pineapple value chain development and covering the Atlantic department, indicated that 92.13% of fresh pineapple producers were present in the five selected municipalities of our study. The adjusted “*n*” was then calculated and was 306 (333 × 92.13%) fresh pineapple producers. Thus, a total of 306 fresh pineapple producers were finally surveyed.

**FIGURE 1:**
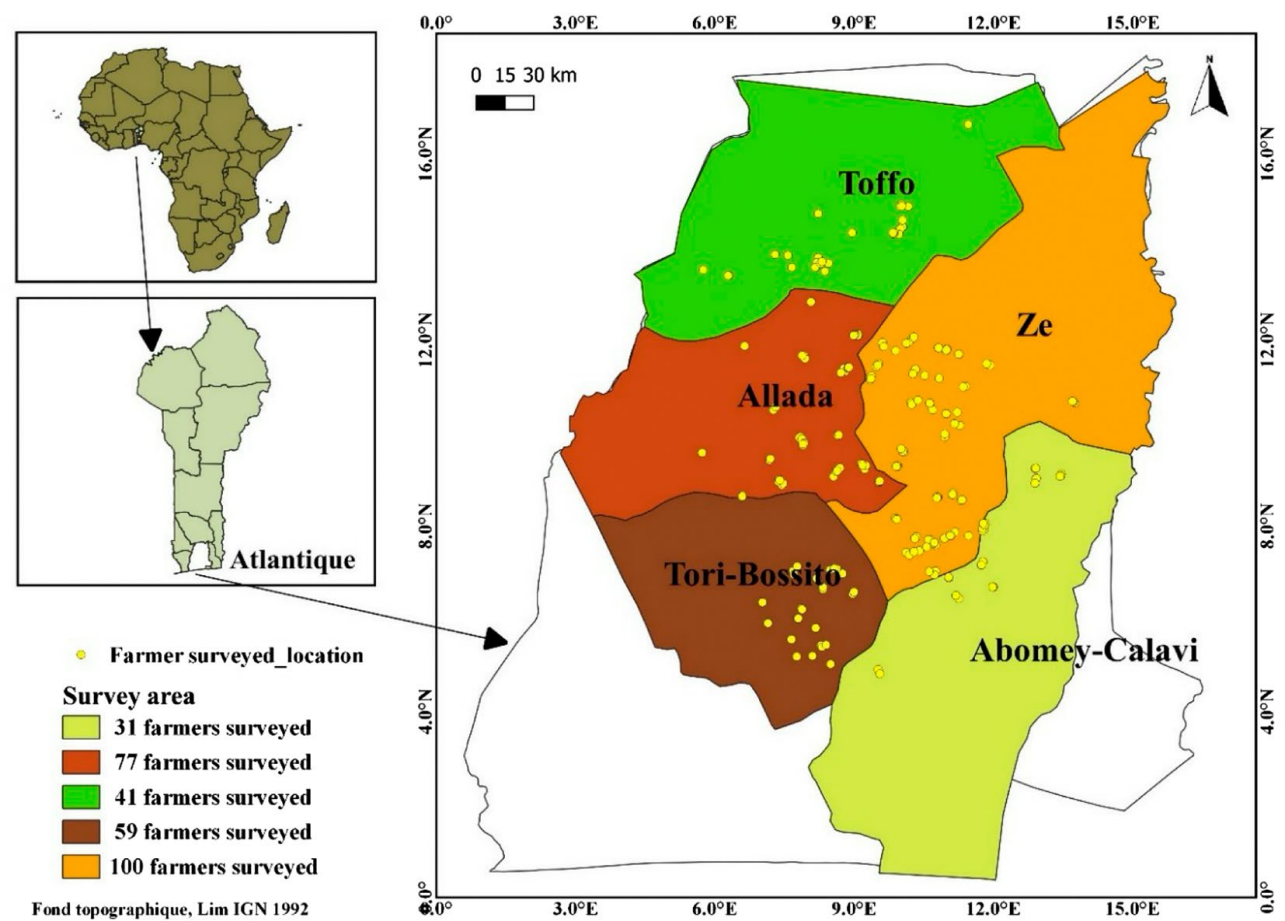
Map depicting fresh pineapple producers surveyed within each of the five municipalities in the Atlantic department, Republic of Benin.

The number of fresh pineapple producers surveyed per municipality “*n*_c_” was determined (Figure 1) proportionally to the number of fresh pineapple producers present in each municipality.

$$n_{c}=\mathrm{Pm}\left(\%\right)\times n$$

With, Pm (%) = Proportion of fresh pineapple producers in the selected municipality (number of fresh pineapple producers/total number of fresh pineapple producers in all five municipalities); *N* = total number of fresh pineapple producers sampled in the Atlantic department; *n*_c_ = number of fresh pineapple producers to be surveyed per municipality.

Fresh pineapple producers were surveyed independently of the pineapple cultivar grown. A semi‐structured questionnaire was developed and tested. Adjustments were made when relevant. The questionnaire (Data S1) was digitized and uploaded onto the platform KoboCollect. Key themes of the questionnaire used for data collection comprised (1) socio‐economic data of respondents, (2) agronomic practices used, and (3) artificial flowering induction practices and perceived effect on health. Verbal informed consent was obtained from all respondents before administering the questionnaire. Data were collected from April to June 2023.

### 2.2 Data Analysis

Descriptive statistics were used to describe the socio‐demographic characteristics of the respondents using R software version 4.3.0 (R Core Team 2023). A chi‐square test was performed to compare the proportion of fresh pineapple producers falling within different categories across the municipalities. Graphs depicting the percentage of respondents in the five selected municipalities who allocated some areas of their land for fresh pineapple production were plotted using R software. In order to describe the agronomic practices used and trends for different practices, percentages were used. Proportions of producers applying a certain product to induce flowering were compared across municipalities using a chi‐square test (Pandis 2016).

## 3 Results

### 3.1 Socioeconomic Characteristics of Fresh Pineapple Producers in the Atlantic Department

In the Atlantic department and across all five surveyed municipalities, men were more involved in fresh pineapple production than women (Table 1). In the Toffo and Tori municipalities, the proportion of women involved tended to be higher than in other municipalities. On average, producers in the Atlantic department were 43 years old. Across all the municipalities, > 50% of producers were < 45 years old. The youngest population of producers was found in the Abomey Calavi municipality, where about 42% were < 35 years old (Table 1). Regarding educational level, most producers in the Atlantic department attended primary and secondary schools. However, literate producers were found in high proportion in Abomey‐Calavi, Toffo, and Tori‐Bossito.

**TABLE 1:** Percentage of fresh pineapple producers with different sociodemographic characteristics in the Atlantic department, Republic of Benin (*n* = 308).

Characteristics	Municipalities						
	Abomey‐Calavi (*n* = 31)	Allada (*n* = 77)	Toffo (*n* = 41)	Tori‐Bossito (*n* = 59)	Zè (*n* = 100)	Atlantic (*n* = 308)	*χ* ^2^ (Chi‐square) *p*
*Gender*							
Male	90.32	92.20	82.93	86.44	96.00	90.90	
Female	09.68	07.79	17.07	13.56	04.00	09.09	
*χ* ^2^ (Chi‐square)	7.18e‐06	1.e‐13	0.126	2.167e‐08	2.2e‐16	2.2e‐16	0.095
*Age*							
≤ 35	41.93	20.78	31.70	32.20	22.00	26.94	
35 to 45	32.26	61.03	36.59	47.46	37.00	44.48	
45 to 55	25.81	16.88	19.51	13.56	26.00	20.45	
55 to 65	00.00	01.30	20.44	01.69	11.00	04.54	
> 65	00.00	00.00	9.76	05.08	04.00	03.57	
*χ* ^2^ (Chi‐square)	0.000144	2.e‐16	0.001997	5.545e‐09	1.0e‐06	2.2e‐16	< 0.001***
*Educational level*							
No formal education	00.00	00.00	00.00	00.00	31.00	10.06	
Literate	51.61	27.27	43.90	50.85	22.00	34.74	
Primary	29.03	45.45	14.63	22.03	25.00	28.57	
Secondary	19.35	20.78	39.02	25.42	21.00	24.02	
University	00.00	06.49	02.44	01.69	01.00	02.59	
*χ* ^2^ (Chi‐square)	7.24e‐06	4.e‐10	6.6e‐07	2.5e‐10	3.8e‐05	2.2e‐16	< 0.001***
*Years of experience*							
≤ 2	9.68	3.90	4.88	1.69	04.00	4.22	
3 to 5	9.68	9.09	26.83	28.81	12.00	16.23	
6 to 10	16.13	40.26	24.39	42.37	22.00	30.19	
11 to 20	45.16	29.87	36.59	25.42	44.00	36.03	
21 to 30	16.13	12.99	7.32	1.69	16.00	11.36	
≥ 30	3.26	3.90	00.00	00.00	02.00	1.94	
*χ* ^2^ (Chi‐square)	0.001102	5.e‐10	8.e‐05	4.9e‐11	8.4e‐14	2.2e‐16	< 0.001***
*Producer association membership*							
Yes	22.58	46.75	65.85	40.68	34.00	41.56	
No	77.42	53.25	34.15	59.32	66.00	58.44	
*χ* ^2^ (Chi‐square)	0.002	0.5688	0.042	0.1521	0.001	0.003	< 0.01**

** Statistically significant at 0.01 > *p* ≥ 0.001.

*** Statistically significant at *p* < 0.001.

Regarding experience in pineapple cultivation, most producers attested to having at least 10 years of experience regardless of the municipality where they grew pineapple. Overall, more than half of the producers surveyed were not a member of a producer's organization. The trend was different in the municipality of Toffo, where 66% of producers were members of an association (Table 1). Approximately 93% of producers reported acquiring cultivated land through rental agreements, while 14% stated that they obtained it through inheritance, 5% through purchase, and 1% through borrowing.

Two fresh pineapple cultivars were grown: cv. Sugarloaf and cv. Smooth Cayenne. Within our sample, the total area devoted to cv. Sugarloaf was 561.64 ha (84% of total area under pineapple cultivation) compared to only 110.30 ha (16% of total area under pineapple cultivation) for cv. Smooth Cayenne. On average, fresh pineapple producers in the Atlantic department allocated 1.82 ha with a range of [0.12–18] ha for cv. Sugarloaf and [0.17–12] ha for cv. Smooth Cayenne.

**FIGURE 2:**
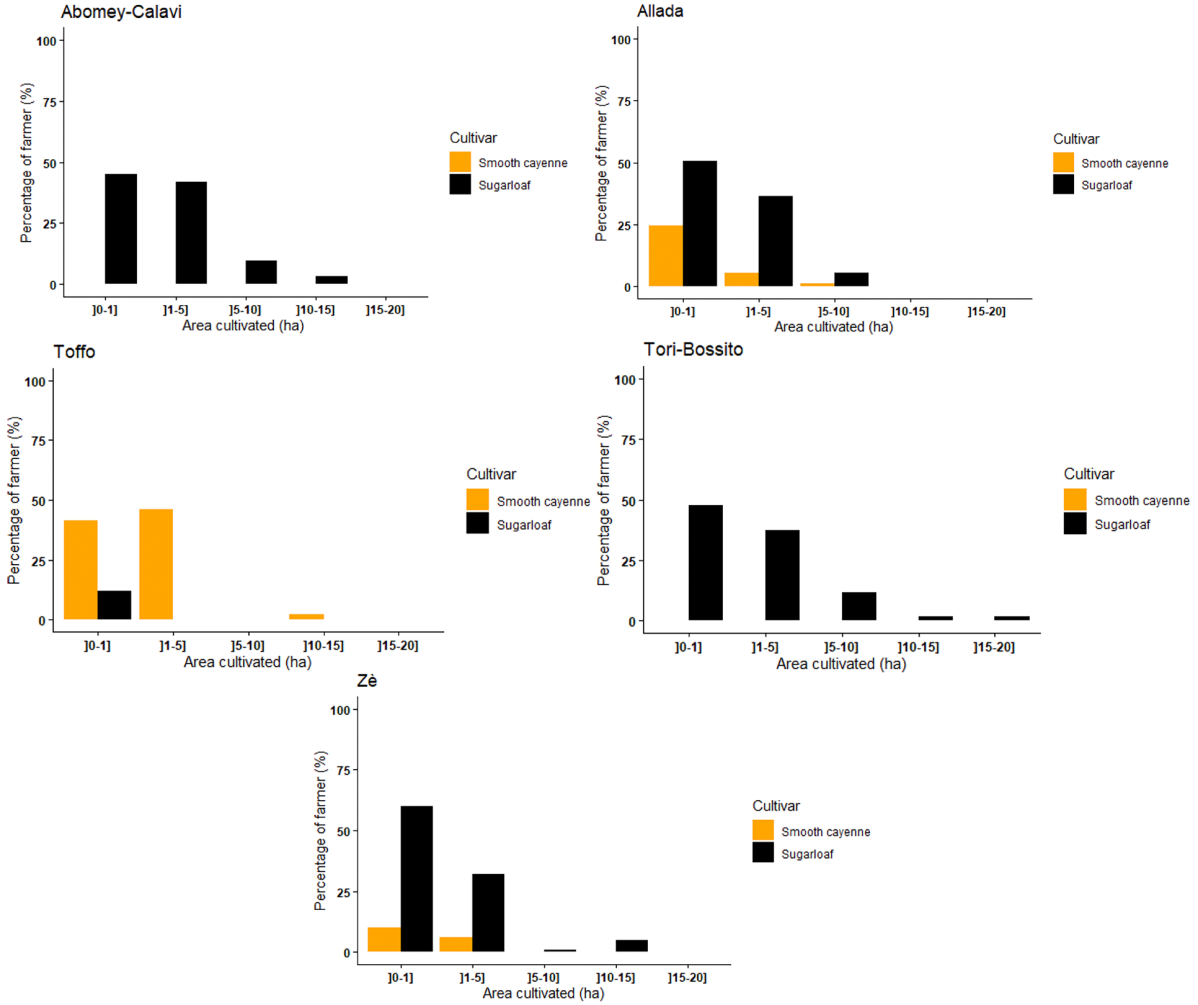
Percentage of fresh pineapple producers allocating a given area of their land for fresh pineapple production in the five selected municipalities in the Atlantic department, Republic of Benin.

From one municipality to another, the tendency of producers to grow a given cultivar differed (Figure 2). Although cv. Sugarloaf was grown in all municipalities, our results showed that all producers surveyed in Abomey‐Calavi and Tori grew solely cv. Sugarloaf, with > 45% of them owning < 1 ha of cv. Sugarloaf and nearly 42% holding 1–5 ha. Regarding cv. Smooth Cayenne, it was mainly produced in the municipality of Toffo, with > 41% of producers surveyed growing it on < 1 ha and > 46% using 1–5 ha. In the municipalities of Allada and Zè, cultivation was dominated by cv. Sugarloaf.

### 3.2 Fresh Pineapple Production Systems in the Atlantic Department

#### 3.2.1 Current Agronomic Practices Used by Fresh Pineapple Producers for cv. Sugarloaf and cv. Smooth Cayenne

Fresh pineapple production is characterized by a series of agronomic practices: plowing, planting, weeding, fertilization, artificial flowering induction, harvesting, and production of planting materials, with some practices being cultivar dependent.

**FIGURE 3:**
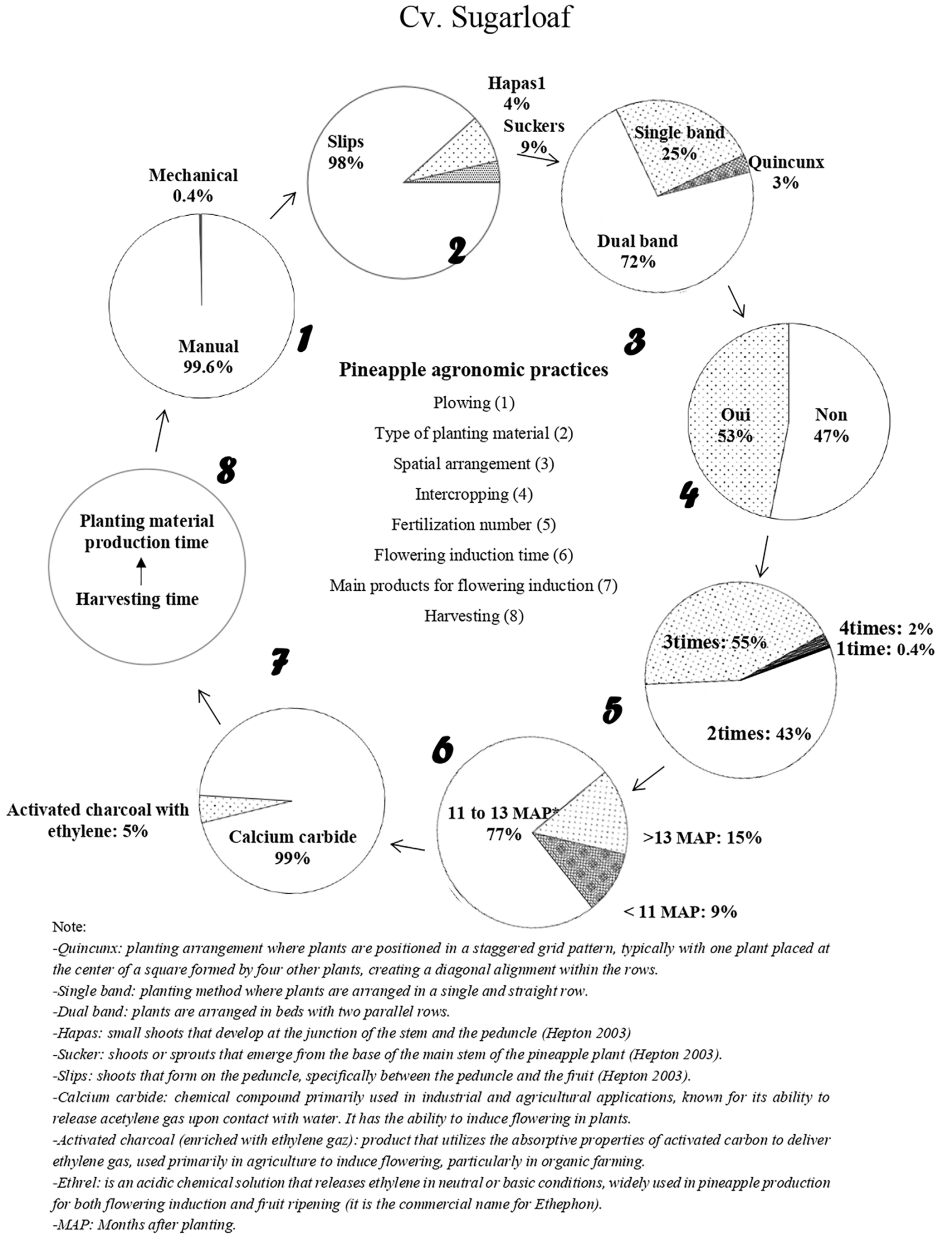
Production cycle of pineapple cv. Sugarloaf in the Republic of Benin.

In cv. Sugarloaf, plowing was done manually by the majority of producers surveyed, who reported the use of slips as planting material. They grew cv. Sugarloaf in double rows (Figure 3). More than half of those surveyed growing cv. Sugarloaf intercropped it with other crops such as maize, cassava, tomato, cowpea, peanut, pepper, and watermelon. The number of fertilizations varied a lot, with most of those surveyed applying chemical fertilizers three times within the production period. Artificial flowering induction took place in most cv. Sugarloaf farms between 11 and 13 months after planting, with calcium carbide being the most used compound. After harvesting, ratoon crops were left to produce planting materials of appropriate size for the next planting round.

**FIGURE 4:**
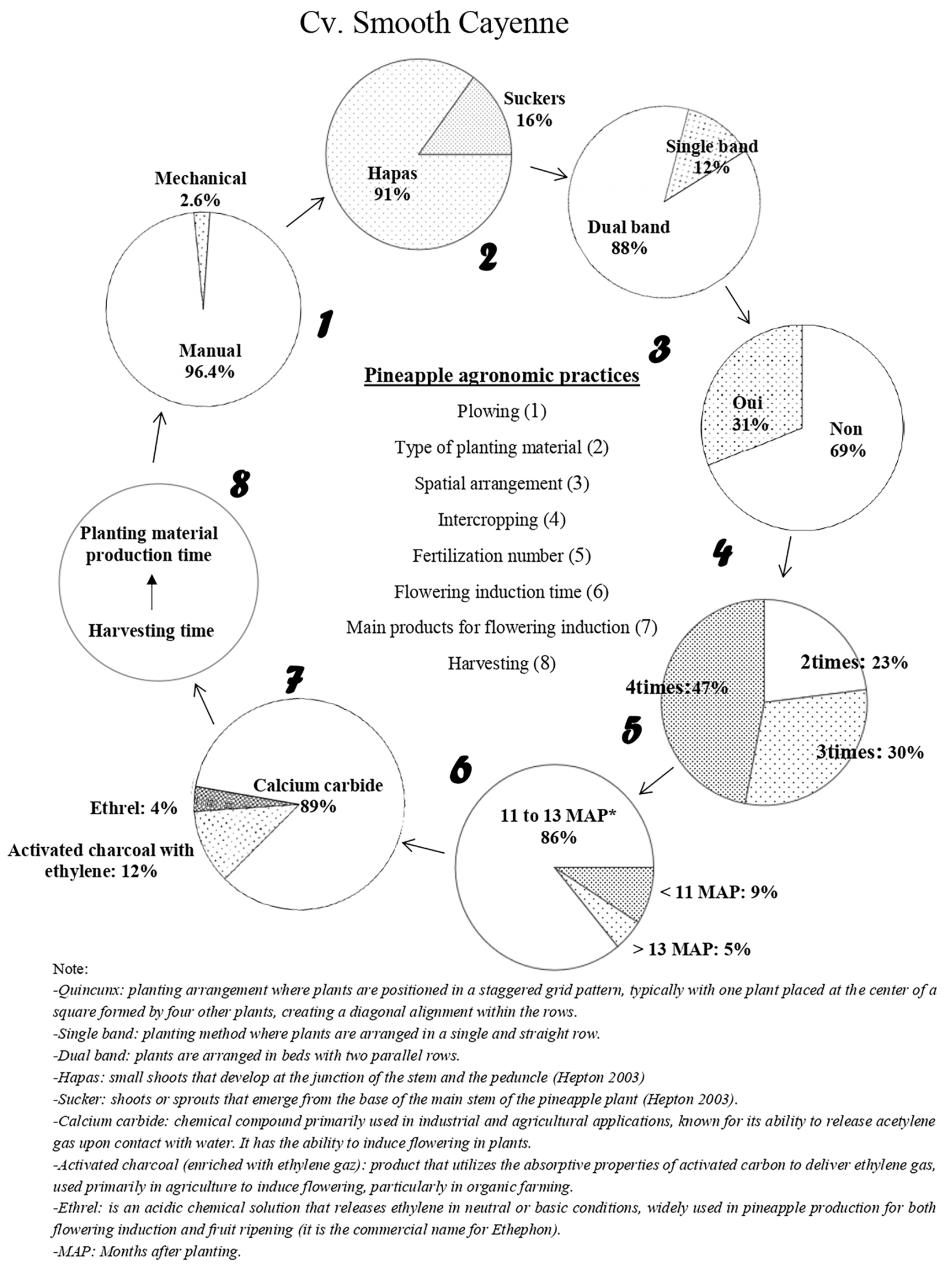
Production cycle of pineapple cv. Smooth Cayenne in the Republic of Benin.

Plowing was also done manually by the majority of those surveyed growing cv. Smooth Cayenne. These producers reported the use of hapas (small shoots that develop at the junction of the stem and the peduncle) as planting material. They grew cv. Smooth Cayenne in double rows (Figure 4). About one‐third of those surveyed growing cv. Smooth Cayenne intercropped it with other crops such as maize, cowpea, tomato, and peanut. The number of fertilizations varied a lot, with most producers fertilizing four times. Artificial flowering induction took place in most cv. Smooth Cayenne farms between 11 and 13 months after planting, with calcium carbide being the most used compound and applied three times at two‐day intervals. After harvesting, ratoon crops were maintained to produce planting materials of appropriate size for the next planting round.

#### 3.2.2 Fertilization Practices in cv. Sugarloaf and cv. Smooth Cayenne Production

There was a huge diversity in pineapple fertilization practices in terms of time of application and type of fertilizers used.

**FIGURE 5:**
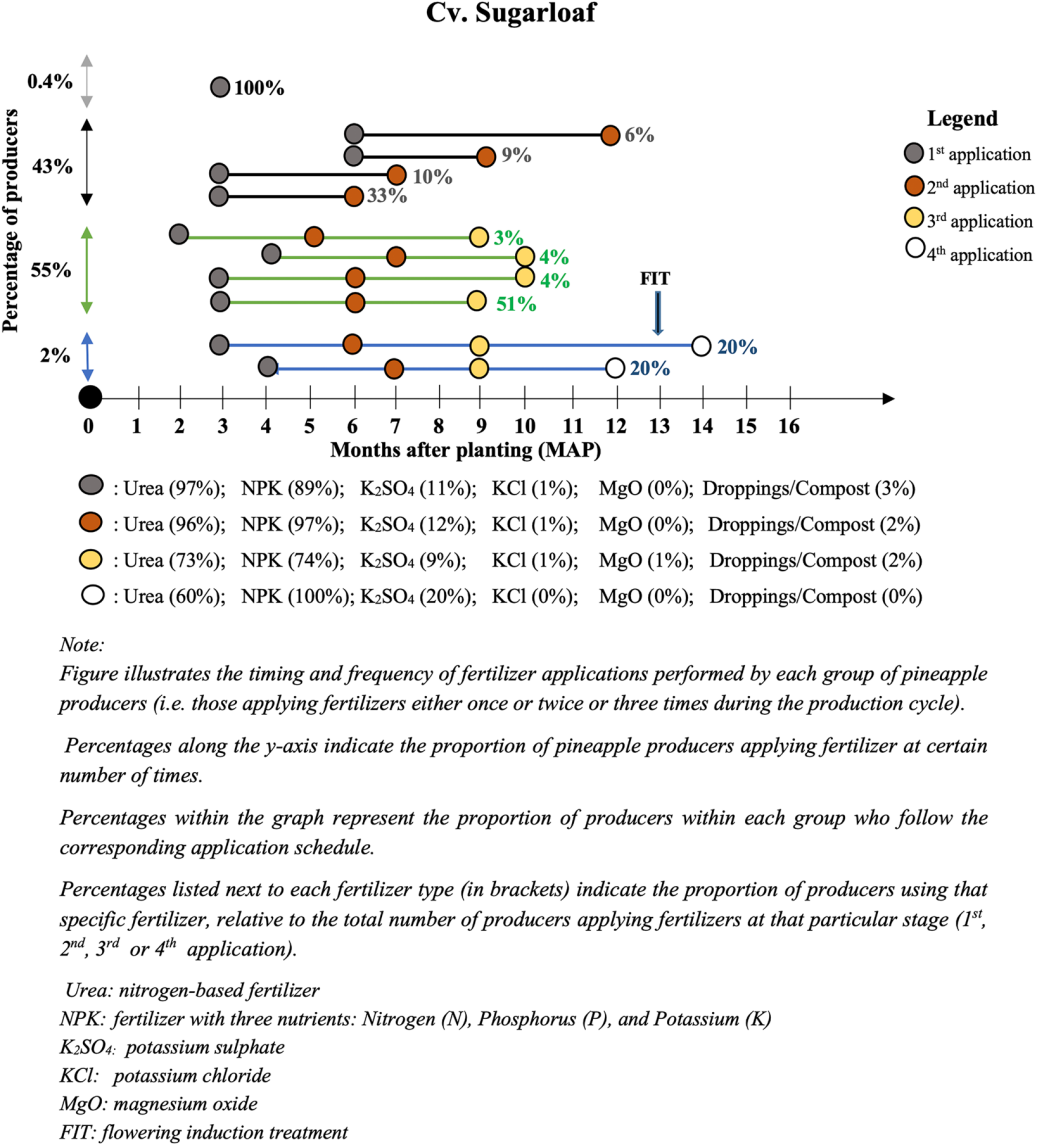
Fertilization practices in cv. Sugarloaf in the Atlantic department of the Republic of Benin.

Cv. Sugarloaf producers applied chemical fertilizers one, two, three or four times during pineapple cultivation. More than half of these respondents confirmed applying fertilizers three times (55%), primarily at 3, 6, and 9 months after planting (Figure 5). 43% of those surveyed applied fertilizers twice and mainly at 3 and 6 months after planting. Only a small number (2%) of those surveyed applied fertilizers four times, typically at 3, 6, 9 and 12 months after planting. In all cases, urea, NPK, or K_2_SO_4_ were the main fertilizers reported.

**FIGURE 6:**
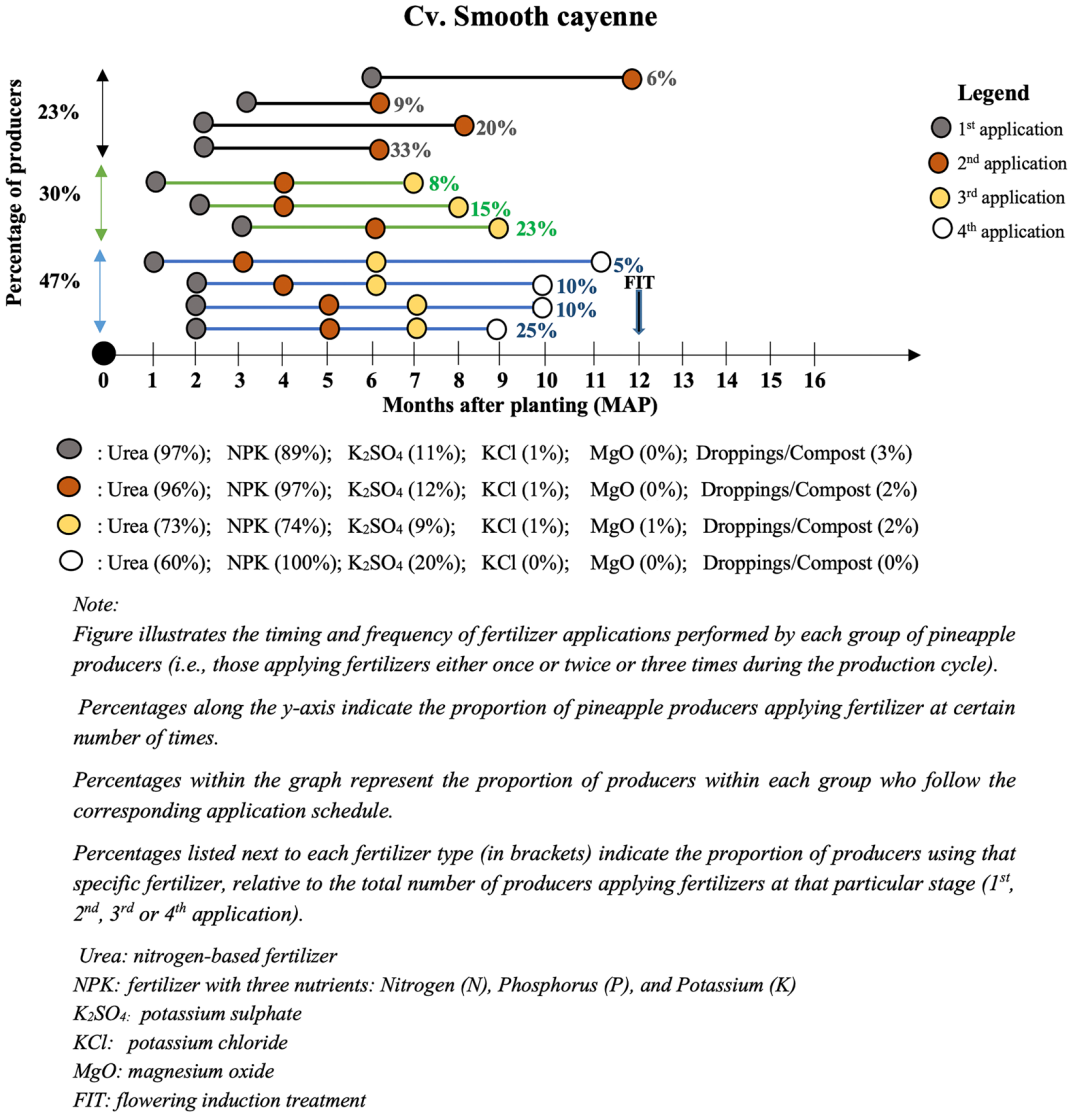
Fertilization practices in cv. Smooth Cayenne in the Atlantic department of the Republic of Benin.

Cv. Smooth cayenne producers applied chemical fertilizers two, three or four times during cultivation. Most of them confirmed applying fertilizers four times (47%), primarily at 2, 6, 7, and 9 months after planting (Figure 6). 30% applied fertilizers three times, primarily at 3, 6, and 9 months after planting. Few of them (23%) applied fertilizers twice and mainly at 2 and 6 months after planting. In all cases, urea, NPK, or K_2_SO_4_ were the main fertilizers reported.

### 3.3 Compounds Used for Artificial Flowering Induction in cv. Sugarloaf and cv. Smooth Cayenne

Overall, cv. Sugarloaf producers used two main types of products: (i) activated charcoal enriched with ethylene gas and (ii) calcium carbide (Tables 2 and 3).

**TABLE 2:** Compounds used for artificial flowering induction of cv. Sugarloaf plants as reported by surveyed respondents in the Atlantic department, Republic of Benin (percentage values).

Compounds used for artificial flowering induction	Abomey‐Calavi (*n* = 31)	Allada (*n* = 71)	Toffo (*n* = 5)	Tori‐Bossito (*n* = 59)	Zè (*n* = 98)	Atlantic Department (*n* = 264)	*χ* ^2^ (Chi‐square) *p*
CaC_2_	80.65	90.57	75.00	77.97	72.62	78.35	0.515
CaC_2_ + P	03.23	05.66	**25.00**	05.08	13.10	13.74	**< 0.001** ***
CaC_2_ + Sg	**12.90**	01.88	00.00	05.08	09.52	11.45	**< 0.001** ***
CaC_2_ + S	00.00	00.00	00.00	00.00	01.19	03.05	0.3128
CaC_2_ + D	00.00	00.00	00.00	00.00	00.00	00.00	0.3128
CaC_2_ + P + S	00.00	00.00	00.00	**05.08**	01.19	03.05	**< 0.05** *
CaC_2_ + P + Sg	00.00	00.00	00.00	01.69	04.76	03.81	**< 0.05** *
CaC_2_ + P + Sg + S	03.23	00.00	00.00	01.69	01.19	02.29	0.3211
CaC_2_ + Sg + U	00.00	01.88	00.00	00.00	00.00	00.43	0.1958
CaC_2_ + H + S	00.00	00.00	00.00	00.00	00.00	00.00	0.3128
Ethrel/Ethephon	00.00	00.00	00.00	00.00	00.00	00.00	0.3128
Activated charcoal with ethylene gas	00.00	03.77	**25.00**	18.64	00.00	05.19	**< 0.001** ***

*Note:* Values in bold indicate the highest proportion of fresh pineapple production for a given treatment combination.

Abbreviations: CaC_2_, calcium carbide; D, Powder detergent; H, Motor oil; P, Petroleum; S, Cooking salt; Sg, Super Gro (an organic liquid fertilizer); U, Urea.

* Statistically significant at 0.05 > *p* ≥ 0.01.

*** Statistically significant at *p* < 0.001.

**TABLE 3:** Compounds used for artificial flowering induction of cv. Smooth Cayenne plants as reported by surveyed respondents in the Atlantic department, Republic of Benin.

Compounds used for artificial flowering induction	Abomey‐Calavi (*n* = 0)	Allada (*n* = 24)	Toffo (*n* = 37)	Tori‐Bossito (*n* = 0)	Zè (*n* = 16)	Atlantic Department (*n* = 77)	*χ* ^2^ (Chi‐square) *p*
CaC_2_	—	95.83	62.16	—	64.70	72.72	**< 0.01** **
CaC_2_ + P	—	04.17	08.11	—	11.76	07.79	**0.1656**
CaC_2_ + Sg	—	00.00	00.00	—	**05.88**	01.29	**< 0.01** **
CaC_2_ + S	—	00.00	**10.81**	—	00.00	05.19	**< 0.001** ***
CaC_2_ + D	—	00.00	00.00	—	**05.88**	01.29	**< 0.01** **
CaC_2_ + P + S	—	00.00	00.00	—	00.00	00.00	0.3128
CaC_2_ + P + Sg	—	00.00	00.00	—	00.00	00.00	0.3128
CaC_2_ + P + Sg + S	—	00.00	00.00	—	00.00	00.00	0.3128
CaC_2_ + Sg + U	—	00.00	00.00	—	00.00	00.00	0.3128
CaC_2_ + H + S	—	00.00	02.70	—	00.00	01.29	0.6721
Ethrel/Ethephon	—	00.00	**08.11**	—	00.00	03.89	**< 0.001** ***
Activated charcoal with ethylene gas	—	04.17	**16.22**	—	11.76	11.69	0.3132

*Note:* Values in bold indicate the highest proportion of fresh pineapple producing for a given treatment combination.

Abbreviations: CaC_2_, calcium carbide; D, Powder detergent; H, Motor oil; P, Petroleum; S, Cooking salt; Sg, Super Gro (an organic liquid fertilizer); U, Urea; —, Not applicable.

** Statistically significant at 0.01 > *p* ≥ 0.001.

*** Statistically significant at *p* < 0.001.

Most cv. Sugarloaf and cv. Smooth Cayenne producers used calcium carbide alone with no additional products. Some mixed the calcium carbide with petroleum, “Super Gro” (an organic liquid fertilizer from NEOLIFE, a private manufacturer and made from poultry droppings, and sea bird guano) or even cooking salt (Tables 2 and 3).

Regarding practices across municipalities, for cv. Sugarloaf, use of calcium carbide combined with petroleum was mainly in the municipality of Toffo; use in combination with Super Gro was prevalent in Abomey‐Calavi. The practice of using calcium carbide combined with petroleum plus cooking salt was mainly observed in Tori‐Bossito, while use in combination with petroleum and Super Gro was mainly in Zè (Table 2).

Regarding cv. Smooth Cayenne, which is only grown in Allada, Toffo, and Zè (Figure 2), the use of calcium carbide combined with Super Gro or detergent powder to induce flowering was prevalent in Zè while the use in combination with cooking salt and Ethrel was observed in Toffo (Table 3).

The use of activated charcoal with ethylene gas to induce pineapple plants regardless of the cultivar grown was mainly observed in Toffo.

Description of how various product combinations used by producers was described in the supplementary information 2 (Table S1).

### 3.4 Perceived Effects of Calcium Carbide on Fresh Pineapple Producers' Health

**FIGURE 7:**
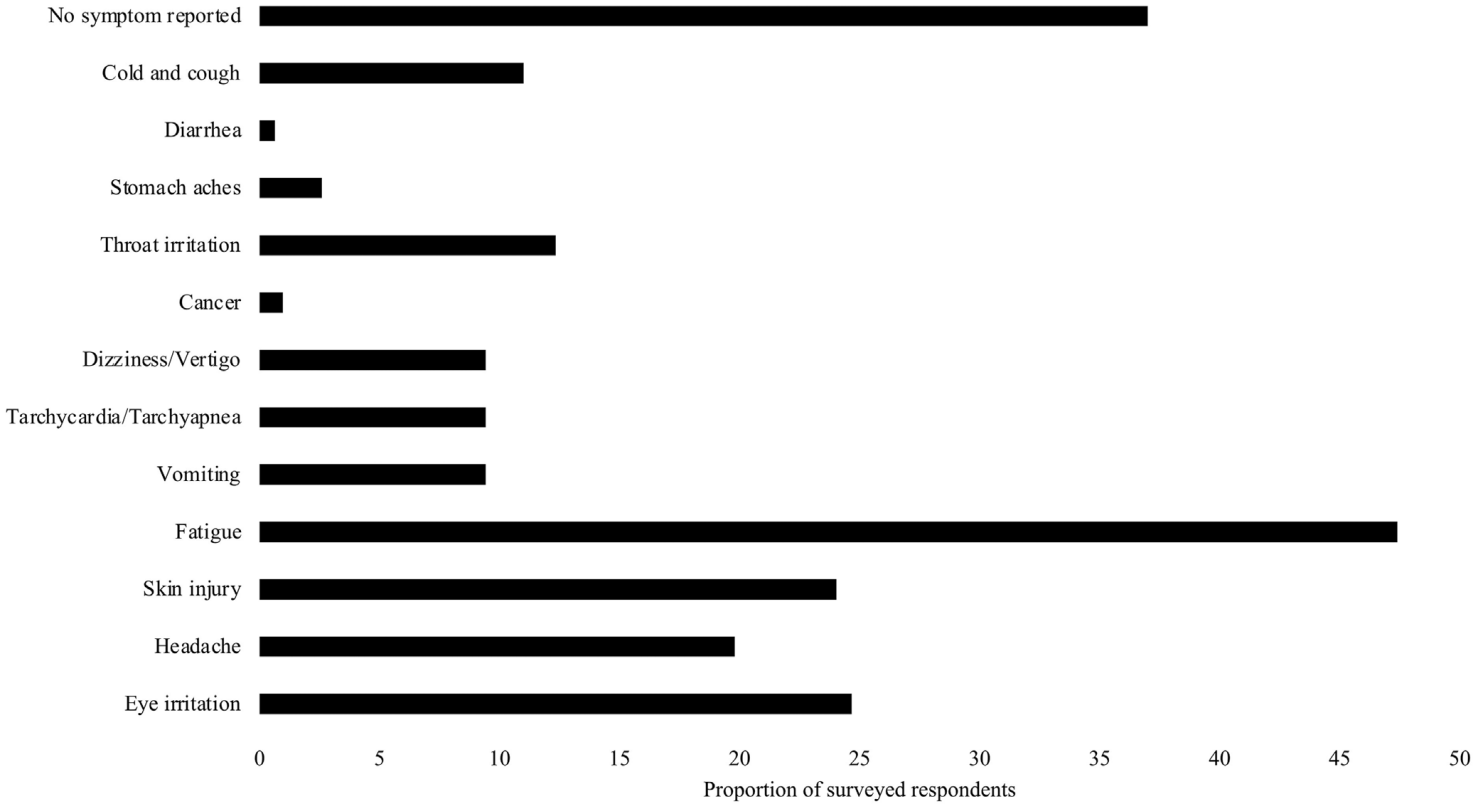
Proportion of fresh pineapple producers reporting symptoms after applying or being in contact with calcium carbide (used for artificial flowering induction) in the Atlantic department, Republic of Benin.

More than half of producers surveyed stated that they were discomforted after using calcium carbide. Reported symptoms varied (Figure 7). The most common symptoms identified included fatigue (47%), eye irritation (25%), skin injury (24%), headache (20%), and cold and cough (11%) (Figure 7). Less common symptoms such as vomiting (9%), tachycardia, or tachypnea and dizziness were also reported, as well as a few cases of cancer and diarrhea. Furthermore, 8% of respondents mentioned that at least one member of their family experienced a health problem following contact or handling of calcium carbide stored at home.

## 4 Discussion

The objective of this paper was to revisit the fresh pineapple production system in the Republic of Benin (Atlantic Department) by analyzing current agronomic practices used by producers. The emphasis was on flowering induction practices and their perceived effects on health.

### 4.1 Socioeconomic Characteristics of Fresh Pineapple Producers in the Atlantic Department: What Has Changed Over the Last 10 Years?

Our results showed that men dominate fresh pineapple production in the Republic of Benin, mainly in the Atlantic department (Table 1). However, there is a clear increase in the proportion of women involved, from 3.39% in 2013 (Achigan‐Dako et al. 2014) to > 9% in 2023 (Table 2). The participation of young people (aged 35 years or less) in pineapple production has decreased, from 60% in 2013 to 26% (Table 1). Compared to 10 years ago, this study revealed a clear improvement in the level of schooling or literacy of fresh pineapple producers, going from a 75% illiteracy rate in 2013 (Sossa et al. 2014) to only 10%. This probably explains the increase in the proportion of producers belonging to at least one producer association, which increased from 10% in 2013 (Sossa et al. 2014) to 42% in 2023 (Table 1).

Land rental remained the main mode of access to land. Referring to the findings of Sossa et al. (2014), our results indicated that the percentage of producers growing their crop on rented areas has increased over the last ten years (80.4% to 93%), while the percentage of producers operating on their own land obtained by inheritance decreased (18.6% to 14%). This might be because, in many pineapple production zones, the government took over lands from producers for infrastructure development. Under these conditions some producers might have lost lands they inherited and, therefore, been obliged to rent land to cultivate their crops. As a result, the profitability of pineapple cultivation could have been negatively impacted by additional costs associated with land rental, reducing overall margins. The pineapple cultivars produced in Benin are still cv. Sugarloaf and cv. Smooth Cayenne. The cv. Sugarloaf remains the most cultivated, with an allocation of 84.2% of land allocated to pineapple production in the Atlantic department and 15.8% for cv. Smooth Cayenne. Ten years ago, studies by Achigan‐Dako et al. (2014) revealed that 79% of producers surveyed exclusively cultivated cv. Sugarloaf, with only 2% engaged in the cultivation of cv. Smooth Cayenne and 18% in both cultivars. Our study showed a slight increase in the interest given to cv. Smooth Cayenne (10.4%). The increase in interest given to cv. Smooth Cayenne could be explained by the relative increase in the export of fresh pineapple fruits from Benin compared to a decade ago. In reference to the same studies by Achigan‐Dako et al. (2014), the production of cv. Smooth Cayenne which was only restricted to two municipalities (Toffo and Zè) in 2013, has now spread to the municipality of Allada (Figure 2). The production of cv. Sugarloaf has significantly decreased in Toffo municipalities over the years. The producers of the municipalities of Abomey‐Calavi and Zè have remained faithful to the cultivation of cv. Sugarloaf during the last decade. This study revealed an increase of very small producers with < 1 ha to the detriment of larger production areas (Figure 2).

### 4.2 Fresh Pineapple Production Systems in the Atlantic Department

Fresh pineapple production practices vary from one cultivar to another (Fassinou Hotegni et al. 2012). In reference to the previous authors, we observed that in the production of both cv. Sugarloaf and cv. Smooth Cayenne, plowing is still done manually. Most cv. Sugarloaf producers (98%) had a preference for the use of slips; cv. Smooth Cayenne producers used hapas and suckers much more but with a pronounced preference for hapas. These results are similar to those obtained by Fassinou Hotegni et al. (2012) and can be explained by the fact that the slips are produced in a large number in cv. Sugarloaf, as reported by Fassinou Hotegni et al. (2014). Consequently, producers growing cv. Sugarloaf can easily harvest them when they reach an appropriate size and use them as planting material for the next season. In the case of cv. Smooth Cayenne, hapas and suckers are the dominant planting materials produced by the crop (Fassinou Hotegni et al. 2012). Some producers intercropped pineapple with other crops such as corn, cassava, tomatoes, peppers, and others. Such decisions might be because pineapple is a long‐cycle crop (15–18 months after planting); producers then grow short‐cycle crops in order to have financial resources for subsistence, as argued by Fassinou Hotegni et al. (2012) and Ajayi et al. (2018). Studies by Custódio et al. (2016) on the benefits of pineapple‐cassava intercropping showed that when intercropped with cassava, pineapple plants yield higher fruit weights and yields compared to those grown in direct sunlight. In addition, cassava crops protect pineapple fruits against sun damage, which negatively affects fruit quality. Uriza‐Ávila, Rebolledo‐Martínez, and Rebolledo‐Martínez (2005) demonstrated that intercropping with corn, tomato, or pepper is beneficial for the short‐cycle crop as pineapple plants help protect them from heavy rainfall. Furthermore, Siebeneichler et al. (2019) revealed that intercropping pineapple with cowpea could be a viable option for farmers in Tocantins state (Brazil) when considering the integration of legumes into the cropping system.

Fertilization practices did not change much in a decade. In their work, Fassinou Hotegni et al. (2012) pointed out that the number of fertilizer applications ranged between one and four in cv. Sugarloaf production and between two and four times in cv. Smooth Cayenne production, with a majority opting for three applications. The trends seem to be the same after 10 years. For cv. Sugarloaf production, the proportion of producers applying three times increased from 36% to 55%. In contrast, for cv. Smooth Cayenne production, the percentage of those who applied three times decreased from 54% to 47%, while the percentage of those who applied four times increased from 6% to 30%. This implies that the quantity of fertilizer required to produce pineapples today compared to a decade ago has increased significantly. Such an increase in the use of fertilizers may lead to higher production costs for fresh pineapple producers along with a huge environmental impact, hence the urgent need for more sustainable and efficient fertilization practices in the future.

The most commonly used types of fertilizers are still NPK, urea, and K_2_SO_4_. This last fertilizer seems to be much more used in the production of cv. Smooth Cayenne, but especially for the last application, indicating that these producers are becoming more aware of the role of potassium in fruit firmness and quality as noted by Cunha et al. (2021).

To enhance pineapple productivity, it would be beneficial to promote sustainable crop management practices. Recent studies in Ghana showed that the use of biochar can help improve yield (Hanyabui et al. 2024).

Artificial flowering induction takes place in most of the cv. Sugarloaf and cv. Smooth Cayenne farms at 11–13 months after planting, with calcium carbide being the main compound used. Producers generally dissolve calcium carbide in water and apply it to the heart of the pineapple plant in order to induce flowering. However, some producers seem to have discovered over the last 10 years a new practice such as adding (i) petroleum or motor oil, (ii) cooking salt, (iii) liquid fertilizer commonly known as “Super Gro”, or (iv) urea to the calcium carbide (Tables 2 and 3, Table S1). They combine these products with the aim of either making the calcium carbide more effective or increasing the size of the pineapple fruits. Some fresh pineapple producers mix several of these products (Tables 2 and 3, Table S1). No study has documented the effect of the addition of these mixtures on the effectiveness of flowering induction or pineapple fruit quality. However, the addition of urea to the calcium carbide can be explained by a strategy used by producers to reduce the paid workforce by combining urea application with flowering induction treatment. Producers using these different practices claim to have adopted them based on advice from friends. Although these changes have only been reported by a few producers (Table 2), they raise questions. Producers who engage in these new, unknown‐origin practices argue that adding these products enhances the induction power of calcium carbide and reduces the failure rate of flowering, even under adverse weather conditions. These findings suggest that further research should be conducted on these alternative and new practices for sustainable artificial flowering induction practices. It also raises the question of whether the use of calcium carbide alone is as effective at the same concentration today as it was 10 years ago or more and highlights the need to develop alternatives to the use of calcium carbide.

### 4.3 Producer's Perceptions in Relation to Different Flowering Induction Compounds

Calcium carbide, due to its explosive nature, is a very dangerous product and difficult to handle. Furthermore, it contains arsenic, which is very carcinogenic (Ikhajiagbe et al. 2022; Okeke et al. 2022). The majority of fresh pineapple producers are aware of the fact that calcium carbide is a dangerous product. However, they do not know to what degree its exposure could be injurious to their health and safety. Our study revealed that most producers used calcium carbide to artificially induce pineapple plants to flower (Tables 2 and 3). Surveyed producers reported discomfort in its application. Symptoms included excessive fatigue, eye irritation, skin injury, cough, cold, and headache. Related common symptoms like vomiting, tachycardia or tachypnea, and dizziness were reported (Figure 7). These results corroborate those of Anani et al. (2020), who revealed that more than half of producers in Togo experienced, at least once, a form of discomfort after using calcium carbide. These mainly involved coughing, skin burning, eye irritation, and some form of abdominal pain. Activated charcoal enriched with ethylene is only used by a few producers (Tables 2 and 3) in the municipality of Toffo. Less than 1% of cv. Sugarloaf producers used Ethrel, while 7% of cv. Smooth Cayenne producers used it. The activated charcoal proposed by COLEAD (Committee Linking Entrepreneurship‐Agriculture‐Development) and recently introduced in Benin represents one of the alternatives to calcium carbide, but this product was reported not to be as effective as the calcium carbide, hence the small number of producers using it. In addition, the use of activated charcoal enriched with ethylene is twice as expensive as that of calcium carbide; consequently, there is little interest in using it, especially among conventional producers. However, it could be a very good option for producers of organic fresh pineapple for more lucrative markets. As per Ethrel (ethephon), it is not well known to producers as being a flowering induction product, rather for fruit ripening. Further research is needed on alternatives to calcium carbide for pineapple plant induction.

## 5 Conclusion

The pineapple production system in the Republic of Benin has experienced some changes over the past 10 years. There has been a slight increase in the participation of women, but a significant decline in the involvement of young people. There is also increasing interest in growing cv. Smooth Cayenne. In terms of agronomic practices, there has been an increase in the amount of fertilizer required for cultivation and a diversification of flowering induction practices, such as the use of calcium carbide combined with numerous products such as cooking salt, petroleum, Super Gro fertilizer, and urea with a huge perceived impact on producers' health. Activated charcoal enriched with ethylene is not widely used. Although positive changes have been observed over the last 10 years, there are still opportunities to improve existing practices and document existing ones through research. There is a need to document and propose efficient and effective alternatives to calcium carbide as used by the majority of producers.

## Conflicts of Interest

The authors declare no conflicts of interest.

## Supporting information


Data S1.

Table S1.

## Data Availability

The datasets generated and analyzed during the current study are available in the Figshare repository: https://doi.org/10.6084/m9.figshare.28029659.
