# Supplementary material for: Fresh Pineapple Agronomy in the Republic of Benin: Recent Trends in Calcium Carbide Use and Producer Perceptions
Source: Plant Environ Interact. 2025 Jan 7;6(1):e70026. doi: 10.1002/pei3.70026 (PMC11707258; doi:10.1002/pei3.70026)
Supplement: Supplementary file 1 — Data S1. [file PEI3-6-e70026-s001.docx]

**Plant-Environment Interactions Supporting Information**

Fresh pineapple agronomy in the Republic of Benin: recent trends in calcium carbide usage and producer perceptions

**Authors:** Nicodème Fassinou Hotegni, Orthia L. F. Linkpon, Charlotte A. O. Adjé_,_ Mouizz A. B. Salaou, Enoch G. Achigan-Dako

**Supplementary information: Questionnaire used for data collection among pineapple producers**

**Questionnaire used for data collection**

**Surveyed actor: Fresh pineapple producers**

**Consent Form**

We are conducting a study to better understand agronomic practices in fresh pineapple production. This research aims to analyze and propose options for improvement. We are particularly interested in:

- The methods you use to grow your pineapples.
- The main compounds or products you use for flowering induction treatments.
- Your perception of the health impacts of these products.

Before we proceed with the questions, we kindly ask for your verbal consent to participate. Participation is entirely voluntary, and you are free to decline or withdraw at any time.

Please note:

- All data collected will be used solely for research purposes.
- Your name and contact information will remain confidential.
- Your data will be securely protected in compliance with applicable privacy regulations.

**Would you like to participate? Yes or No**. *If your answer is "No," we will not proceed with the survey.*

**Please write here the answer of the respondent:**

Name of enumerator:

Date of survey:

**I-Identification of the producer/Respondent**

| Questions | Modalities |
| --- | --- |
| **Q1**-Department (Should be Atlantic) |  |
| **Q2**- Municipality | 1. ABOMEY-CALAVI 2. ALLADA 3. TOFFO 4. TORI-BOSSITO 5. ZÈ |
| **Q3**- Area within the Municipality (Arrondissement) |  |
| **Q4**-Village |  |
| **Q5**-Surmane of the producer |  |
| **Q6**-First name of the producer |  |
| **Q7**-Gender of the producer | 1. Female 2. Male 3. Do not want to disclose |
| **Q8**-Age of the producer (add the exact age of the producer if known, if not, please tick the class category) | 1. ≤ 35 2. 35 to 45 3. 45 to 55 4. 55 to 65 5. ≥ 65 |
| **Q9.** Educational level of the producer | 1-No formal education  2-Literate  3-Primary school  4-Secondary school  5-University level |
| **Q10**-For how many years have you been growing pineapple? |  |
| **Q11**- What is the total area of your farm? |  |
| **Q12. How did you get the land you are farming?** | 1-Rental agreements  2-Inheritance  3-Purchase  4-Borrowing  5-Other (please specify) |
| **Q13**.What is the total area under pineapple production |  |
| **Q14**. What type of fresh pineapple are you cultivating? | 1-cv Sugarloaf  2-cv. Cayenne lisse  3-Both |
| **Q15**. What type of fresh pineapple production are you doing for cv. Sugarloaf? | 1-Organic production  2-Conventional production  3-Both |
| **Q16**. What type of fresh pineapple production are you doing for cv. Smooth Cayenne? | 1-Organic production  2-Conventional production  3-Both |
| **Q17.** What is the cultivated are under cv. Sugarloaf? |  |
| **Q18**. What is the cultivated are under cv. Smooth Cayenne? |  |
| **Q19**-Are you a member of a producer association? | 1-Yes  2-No |
| If so, which one? Please give the name of the organization, what it does and the its location |  |

**II- Agronomic practices for cv. Sugarloaf production**

| **S1**- How do you decide on when to plant your pineapple cv. Sugarloaf? |  |
| --- | --- |
| **S2**: How do you plow the land? | 1. Manual 2. Mechanical |
| **S3**: What type of planting material do you use? | 1. Slips 2. Hapas 3. Suckers 4. Crown |
| **S4**: From where do you source the planting materials? |  |
| **S5**- What kind of planting arrangement do you use? | 1-Simple bands  2-Quincunx  3-Dual bands  4-Other |
| **S6**- Do you intercrop pineapple with other crops? | 1-Yes  2-No |
| *S6-1. If yes, what are the crops you intercrop pineapple with? List them please* |  |
| *S6-2. If No, why you do not intercrop pineapple with other crops?* |  |
| **S7**-How often do you fertilize the pineapple plants over the whole production cycle? | 1-Once  2-Twice  3-Three times  4-Four times  5-Five times  6-More than five times |
| *S-7-1. If once, when does it take place (put number of months after planting) and what type of fertilizer do you use?*  *For the enumerator:* Please put the number of month and specify the type of fertilizer being either Urea, NPK, K_2_SO_4_ etc. |  |
| *S-7-2. If two times, when does it take place (put number of months after planting), and what types of fertilizers do you use?*  *For the enumerator:* Please put the number of month and specify the type of fertilizer being either Urea, NPK, K_2_SO_4_ etc. |  |
| *S-7-3. If three times, when does it take place (put number of months after planting), and what types of fertilizers do you use?*  *For the enumerator:* Please put the number of month and specify the type of fertilizer being either Urea, NPK, K_2_SO_4_ etc. |  |
| *S-7-4. If four times, when does it take place (put number of months after planting), and what types of fertilizers do you use?*  *For the enumerator:* Please put the number of month and specify the type of fertilizer being either Urea, NPK, K_2_SO_4_ etc. |  |
| *S-7-5. If five times, when does it take place (put number of months after planting), and what types of fertilizers do you use?*  *For the enumerator:* Please put the number of month and specify the type of fertilizer being either Urea, NPK, K_2_SO_4_ etc. |  |
| **S8** - When do you apply the artificial Floral Induction Treatment? (and how often)  *For the enumerator:* Specify the number of months after planting. How often means whether the producer induce one times or two times. |  |
| **S9.** What are your criteria to decide on when to artificially induce the pineapple plants? |  |
| **S10.** What are the compounds used to induce the flowering (list them)  *For the enumerator:* Please list them |  |
| **S11**- Could you please describe how you induce the flowering of the compounds listed in S10?  *For the enumerator:* Information on quantity of products used, mixture and number of plants induced is needed here |  |
| **S12**. What period of the day do you induce the flowering? | 1-Morming  2-Mid day  3-Afternoon  4-Evening |
| **S13**. How do you come to know such practice? | 1. Vulgarization (specify which structure) 2. Personal experience 3. Empirical knowledge 4. Advice from other producers |
| **S14**. Who is in charge of the artificial induction practice? | 1-Myself  2-A member of my family  3-Hired labour |

**III- Agronomic practices for cv. Smooth Cayenne production**

| **C1**- How do you decide on when to plant your pineapple cv. Smooth Cayenne? |  |
| --- | --- |
| **C2**: How do you plow the land? | 1. Manual 2. Mechanical |
| **C3**: What type of planting material do you use? | 1. Slips 2. Hapas 3. Suckers 4. Crown |
| **C4**: From where do you source the planting materials? |  |
| **C5**- What kind of planting arrangement do you use? | 1-Simple bands  2-Quincunx  3-Dual bands  4-Other |
| **C6**- Do you intercrop pineapple with other crops? | 1-Yes  2-No |
| *C6-1. If yes, what are the crops you intercrop pineapple with? List them please* |  |
| *C6-2. If No, why you do not intercrop pineapple with other crops?* |  |
| **C7**-How often do you fertilize the pineapple plants over the whole production cycle? | 1-Once  2-Twice  3-Three times  4-Four times  5-Five times  6-More than five times |
| *C-7-1. If once, when does it take place (put number of months after planting) and what type of fertilizer do you use?*  *For the enumerator:* Please put the number of month and specify the type of fertilizer being either Urea, NPK, K_2_SO_4_ etc. |  |
| *C-7-2. If two times, when does it take place (put number of months after planting), and what types of fertilizers do you use?*  *For the enumerator:* Please put the number of month and specify the type of fertilizer being either Urea, NPK, K_2_SO_4_ etc. |  |
| *C-7-3. If three times, when does it take place (put number of months after planting), and what types of fertilizers do you use?*  *For the enumerator:* Please put the number of month and specify the type of fertilizer being either Urea, NPK, K_2_SO_4_ etc. |  |
| *C-7-4. If four times, when does it take place (put number of months after planting), and what types of fertilizers do you use?*  *For the enumerator:* Please put the number of month and specify the type of fertilizer being either Urea, NPK, K_2_SO_4_ etc. |  |
| *C-7-5. If five times, when does it take place (put number of months after planting), and what types of fertilizers do you use?*  *For the enumerator:* Please put the number of month and specify the type of fertilizer being either Urea, NPK, K_2_SO_4_ etc. |  |
| **C8** - When do you apply the artificial Floral Induction Treatment? (and how often)  *For the enumerator:* Specify the number of months after planting. How often means whether the producer induce one times or two times. |  |
| **C9.** What are your criteria to decide on when to artificially induce the pineapple plants? |  |
| **C10.** What are the compounds used to induce the flowering (list them)  *For the enumerator:* Please list them |  |
| **C11**- Could you please describe how you induce the flowering of the compounds listed in S10?  *For the enumerator:* Information on quantity of products used, mixture and number of plants induced is needed here |  |
| **C12**. What period of the day do you induce the flowering? | 1-Morming  2-Mid day  3-Afternoon  4-Evening |
| **C13**. How do you come to know such practice? | 1. Vulgarization (specify which structure) 2. Personal experience 3. Empirical knowledge 4. Advice from other producers |
| **C14**. Who is in charge of the artificial induction practice? | 1-Myself  2-A member of my family  3-Hired labour |

**IV- Perceived effects of calcium carbide on producer health**

| **P1**- Have you been a victim of poisoning or discomfort occurring after application of calcium carbide? Ex: headaches, skin irritation, or death? | 1. Yes 2. No |
| --- | --- |
| **P2-** Has a member of your family been a victim of poisoning or discomfort occurring after application of calcium carbide? Ex: headaches, skin irritation, or death? | 1. Yes 2. No |
| **P2**- Please list the discomfort you experienced after application of calcium carbide to induce flowering in pineapple plants | 1. Eye irritation 2. Headaches 3. Skin injury 4. Nausea / Vomiting 5. Tachycardia and or tachypnea 6. Vertigo / dizziness 7. Cancer 8. Throat irritation 9. Stomach ache 10. Diarrhea 11. Other (please specify) |
| **P3**. In case the producer does not use calcium carbide, please ask him why. |  |

**V. Comments from the producer/respondent**

**………………………………………………………………..………………………………………………………………..………………………………………………………………..………………………………………………………………..………………………………………………………………..………………………………………………………………..………………………………………………………………..………………………………………………………………..………………………………………………………………..………………………………………………………………..………………………………………………………………..………………………………………………………………..………………………………………………………………..………………………………………………………………..………………………………………………………………..**
