# Supplementary material for: Fresh Pineapple Agronomy in the Republic of Benin: Recent Trends in Calcium Carbide Use and Producer Perceptions
Source: Plant Environ Interact. 2025 Jan 7;6(1):e70026. doi: 10.1002/pei3.70026 (PMC11707258; doi:10.1002/pei3.70026)
Supplement: Supplementary file 2 — Table S1. [file PEI3-6-e70026-s002.docx]

**Plant-Environment Interactions Supporting Information**

Fresh pineapple agronomy in the Republic of Benin: recent trends in calcium carbide usage and producer perceptions

**Authors:** Nicodème Fassinou Hotegni, Orthia L. F. Linkpon, Charlotte A. O. Adjé_,_ Mouizz A. B. Salaou, Enoch G. Achigan-Dako

**Supplementary information: Table S1**

| **Table S1 :** Description of approach used by fresh pineapple producers in mixing different compounds for artificial flowering induction in pineapple plants in the Republic of Benin (Quantity of different products based on 3000 pineapple plants) | |
| --- | --- |
| Compounds used for artificial flowering induction of pineapple plants | Approach used to artificially induce flowering in pineapple plants (for 3000 pineapple plants) |
| **CaC_2_** | 1 to 2 kg of calcium carbide in 250 liters of water |
| **CaC_2_+P** | 1 to 2 kg of calcium carbide mixed with ¼ liter of petroleum in 250 liters of water |
| **CaC_2_+Sg** | 1 to 2 kg of calcium carbide mixed with ¼ liter of ‘’Super Gro’’ in 250 liters of water |
| **CaC_2_+S** | 1 to 2 kg of calcium carbide mixed with ½ kg of salt in 250 liters of water (sodium chloride) |
| **CaC_2_+D** | 1 to 2 kg of calcium carbide mixed with ¼ kg of detergent in powder in 250 liters of water |
| **CaC_2_+P+S** | 1 to 2 kg of calcium carbide mixed with ¼ liter of petroleum and ½ kg of salt in 250 liters of water |
| **CaC_2_+P+Sg** | 1 to 2 kg of calcium carbide mixed with ¼ liter of petroleum and ¼ liter of ‘’Super Gro’’ in 250 liters of water |
| **CaC_2_+P+Sg+S** | 1 to 2 kg of calcium carbide mixed with ¼ of liter of petroleum, ¼ liter of ‘’Super Gro’’and ½ kg of salt in 250 liters of water |
| **CaC_2_+Sg+Urea** | 1 to 2 kg of calcium carbide mixed with ¼ liter of ‘’Super Gro’’and ½ kg of urea in 250 liters of water |
|  | 1 kg of calcium carbide mixed with ¼ to ½ and ¼ kg of salt liter of petroleum in 200 liters of water |
| **Activated charcoal with ethylene gas** | 384-560 g in 250 liters of water. |
| CaC_2_: calcium carbide  D: Powder detergent  H: Motor oil  P: Petroleum  Sg: Super Gro (an organic liquid fertilizer)  S: Cooking salt | |
